# Ileocecal intussusception secondary to giant ileal lipoma in patient with chronic constipation: a case report

**DOI:** 10.1097/MS9.0000000000003101

**Published:** 2025-03-07

**Authors:** Maaz Bin Badshah, Qaisar Ali Khan, Marium Aisha, Laila Hassan, Haadi M. Khan, Ravina Verma

**Affiliations:** a Shifa International Hospital, Islamabad, Pakistan; b Khyber Teaching Hospital, MTI KTH, Peshawar, Pakistan; c Bhatti Hospital and Maternity Home, Sukkur, Pakistan; d Lady Reading Hospital, Peshawar, Pakistan; e St. George’s University School of Medicine, True Blue, Granada

**Keywords:** case report, endoscopy, ileocecal intussusception, Intussusception, lipoma

## Abstract

**Introduction:**

Intussusception in adults is rare, comprising 5% of cases, and is often associated with pathological lesions like polyps or tumors. This report highlights a case of ileocolic intussusception due to a giant terminal ileal lipoma incidentally found during a colonoscopy.

**Case presentation:**

A 53-year-old male presented with chronic constipation, mild abdominal pain, and rectal bleeding for 2 years. The patient used laxatives in the past for constipation but it did not improve. A colonoscopy revealed protrusion of the small intestine through the ileocecal valve into the cecum with a 3 cm lipoma as the leading point. A CT scan of the abdomen was performed, revealing findings suggestive of intussusception. The patient underwent laparotomy, the lipoma was excised, and the intussusception was resolved.

**Clinical discussion:**

Symptoms of intestinal lipoma vary widely, from acute obstruction to incidental imaging findings. Surgical intervention is typically necessary to address the underlying cause, such as gastrointestinal lipomas found predominantly in the right colon. The causes of intussusception in patients, such as lipoma, tumor, or polyp, can be found accidentally during routine colonoscopy.

**Conclusion:**

Intussusception in adults can present with chronic constipation and mild abdominal discomfort.

## Introduction

Intussusception is characterized by invading a proximal segment of the bowel into an adjacent distal segment, commonly occurring in children under three years of age. In adults, intussusception is extremely rare, constituting approximately 5% of all intussusceptions and accounting for 1% of all cases of intestinal obstruction^[1]^. Early diagnosis in adults is challenging due to the nonspecific nature of signs and symptoms, which may present over a chronic, sub-acute, or acute course, unlike pediatric intussusception. Adult intussusception symptoms include nausea, vomiting, gastrointestinal bleeding, changes in bowel habits, and abdominal distension or obstruction^[2]^.

**Highlights**

- Intussusception in adults can present with chronic constipation and mild abdominal discomfort.
- The causes of intussusception in patients, such as lipoma, tumor, or polyp, can be found accidentally during routine colonoscopy.
- Symptoms vary widely from acute obstruction to incidental findings on imaging.
- Surgical intervention is typically necessary to address the underlying cause, such as gastrointestinal lipomas found predominantly in the right colon.

Intussusception in adults can be classified based on its location: entero-enteric, colo-colic, ileo-colic, and ileo-caecal. The etiology, clinical presentation, and management of adult intussusception differ significantly from those in children. Adult intussusception is often associated with a pathological lesion acting as a lead point, such as a benign polyp, enlarged mesenteric lymph node, lipoma, Meckel’s diverticulum, lymphoma, gastrointestinal stromal tumor, or primary or metastatic adenocarcinoma^[3]^. It often has an acute nature when associated with intestinal obstruction, typically presenting in an emergency setting. Without obstruction, symptoms may be absent, and intussusception might be diagnosed incidentally through colonoscopy or radiological imaging. In up to 90% of cases, the causal factor can be identified through imaging or surgical specimens^[3]^.

Gastrointestinal tract (GIT) lipomas, although rare, are benign tumors that can serve as a lead point for intussusception. They usually present as sessile polypoid masses in the right colon and are rarely pedunculated. Consequently, adult intussusception requires surgical intervention to remove the lead point along with the associated lesion^[4]^. This case report highlights a case of ileocecal intussusception in adult patients due to a giant lipoma in the terminal ileum as a lead point. The work has been done following the surgical case report (SCARE; Supplementary Digital Content, http://links.lww.com/MS9/A754) guidelines^[5]^.

## Case report

**Figure 1.**
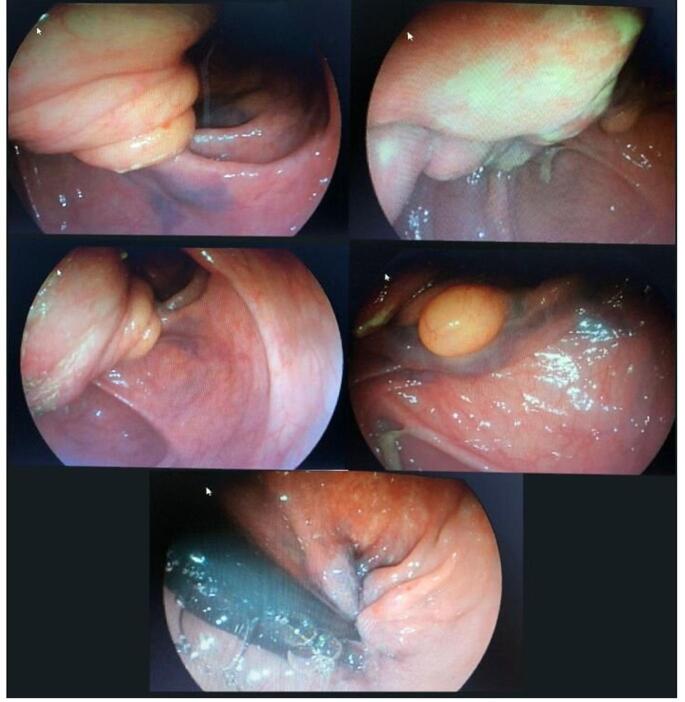
Colonoscopy images showing ileocecal intussusception with 3 cm giant lipoma as a leading point and medium to large hemorrhoids.

A 53-year-old male patient presented to the gastroenterology clinic with complaints of chronic constipation, mild abdominal pain, and rectal bleeding. His symptoms had persisted for 2 years and were initially relieved with laxative use, but the constipation had recently become unresponsive to laxatives. The patient also reported decreased appetite, anorexia, and undocumented weight loss. There was no history of dyspepsia, vomiting, or hematemesis, and his family history was unremarkable for gastrointestinal diseases. The patient was well-oriented to time, person, and place on examination. His vital signs were within normal limits, with a blood pressure of 130/80 mm Hg, a pulse rate of 74 beats per minute, and a temperature of 98.0°F. The abdominal examination revealed a soft abdomen with mild tenderness in the right lower quadrant. The patient’s initial laboratory tests were ordered the results of which are shown in Table 1, and were counseled for colonoscopy due to the alarming signs of weight loss, bleeding per rectum, and chronic constipation. Colonoscopy of the patient was performed under sedation with an intravenous injection of midazolam 4 mg and an injection of nalbuphine 4 mg IV stat. The scope was advanced to the terminal ileum and retroflexed in the rectum. The colonoscopy showed fair bowel preparation and medium to large internal hemorrhoids. There was evidence of a small bowel protrusion through the ileocecal valve into the cecum, with a 3 cm lipoma as a leading point, suggestive of ileocecal intussusception. The rest of the colonic examination was normal, with no bleeding ulcers observed as shown in Fig. 1.

**Figure 2.**
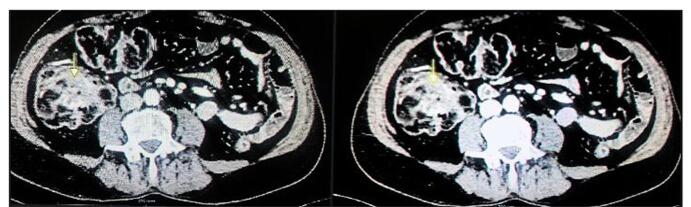
A computed tomographic (CT) scan of the abdomen with contrast showing telescoping of ileum into the cecum suggestive of ileocecal intussusception as indicated by the yellow arrows.

Ultrasound of the abdomen and pelvis was done showing mild fatty liver. A computed tomographic (CT) scan of the abdomen with both intravenous and oral contrast was performed, revealing findings suggestive of the ileocolic type of intussusception with ileum seen telescoping into the cecum and ascending colon up to the hepatic flexure with associated submucosal fatty infiltration and mild mural thickening. Normal enhancing mesenteric vessels with the intussusception as mentioned earlier as shown in Fig. 2.

**Figure 3.**
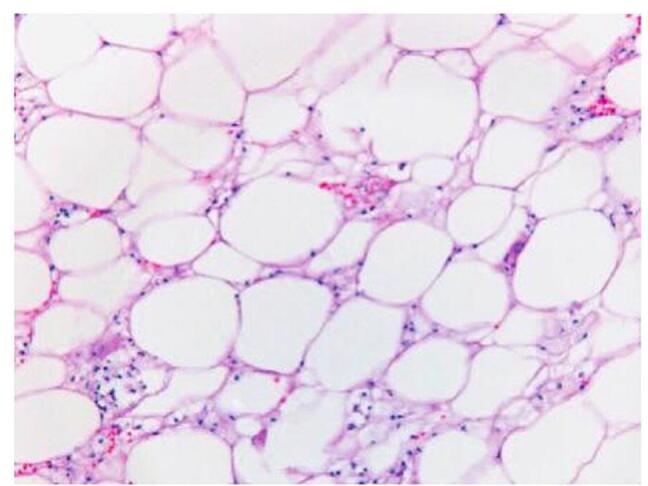
Gross appearance of the excised lipoma.

**Figure 4.**
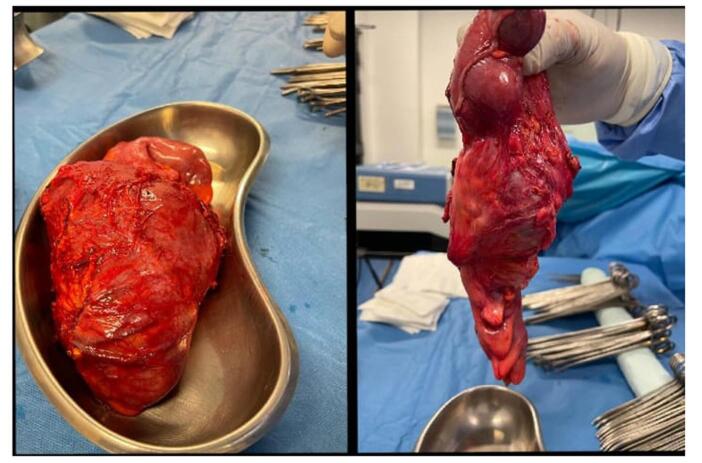
Microscopic examination of the resected specimen showing mature fat cells with no malignant features.

The patient was referred to the general surgery team for further management. A laparotomy was performed under general anesthesia. Right hemicolectomy was done, the lipoma was excised through a formal excision, and stapled anastomosis was done. The excised lipoma is shown in Fig. 3. The terminal ileum biopsy confirmed the presence of a lipoma, showing mature fat cells with no malignant features, as shown in Fig. 4. The patient was discharged home with Ispaghula husk, a tablet of paracetamol 500 mg to be taken as needed, and a tablet of amoxicillin plus clavulanic acid 1 g twice daily for 5 days. At the 8-week follow-up in the gastroenterology clinic, the patient’s constipation had resolved, and he was continued on Ispaghula husk for maintenance.

## Discussion

The exact mechanism of this invagination in adults remains unknown in approximately 20% of cases and is more frequently observed in the small intestine. However, secondary intussusception is thought to originate from any pathological lesion of the intestinal wall that disrupts normal peristaltic activity and acts as a lead point, initiating the invagination of one intestinal segment into another^[3,4,6]^. Intussusception without a lead point is classified as primary or idiopathic, whereas secondary intussusception involves an identifiable lead point. In adults, the most common cause of intussusception is a malignant lesion (65%–75%), followed by benign lesions such as lipomas, polyps, or lymphangiomas (15%–20%)^[7]^. In our case, a lipoma of the terminal ileum served as the lead point for the intussusception, with no malignant features.

In adults, symptoms of intussusception are typically chronic and seldom manifest as an acute abdomen, unlike in children. Most adults experience nonspecific and intermittent abdominal pain, nausea, vomiting, changes in bowel habits, and abdominal distension. Other possible symptoms include melena, fever, and weight loss. Due to the nonspecific and vague nature of these symptoms, preoperative diagnosis is often challenging, necessitating the use of imaging modalities for definitive diagnosis^[7,8]^.

Lipomas are the second most prevalent type of benign gastrointestinal neoplasm, following adenomas in terms of incidence rate. Generally asymptomatic, lipomas larger than 2 cm can cause bowel obstruction, abdominal cramps, bleeding, diarrhea, or lead to intussusception by forming a lead point. These symptoms prompt diagnosis of colonic lipomas using barium enema, abdominal computed tomography, or colonoscopy. Colonoscopy and biopsy are particularly effective diagnostic tools^[6-8]^.

Colonic submucosal lipomas exhibit distinctive features on endoscopy that differentiate them from other submucosal tumors such as gastrointestinal stromal tumors. One characteristic is the “pillow sign, “where the lesion appears soft and cushion-like when pressed with endoscopic forceps. Another is the” naked fat sign,” where fat extrudes after biopsy. Smaller lipomas (less than 2 cm in diameter) and those with a pedunculated morphology and thin stalks are typically removed endoscopically. Surgical resection is often recommended for larger lipomas (greater than 2 cm in diameter) to remove the lead point. In the present case, the lipoma originated from the terminal ileum, measured approximately 3 cm in diameter, and was surgically removed^[9]^.

Azar and Berger, in a retrospective study over 30 years at Massachusetts Hospital in Boston, identified 14 cases of colonic intussusception in adults, three of which were associated with a lipoma^[10]^. Previously reported details of the cases of Ileocecal intussusception secondary to lipoma in an adult are presented in Table 2.

**Table 2:** Literature search

Study	Patient age	Clinical presentation	Diagnostic finding	Treatment	Outcome
Cordeiro J (2019)^[11]^	63	Diffuse abdominal pain, with diarrhea, vomiting and weight loss, in addition to sporadic episodes of hematochezia	Colonoscopy revealed a vegetative-infiltrative lesion, with irregular contours, hardened consistency	Surgery	The patient progressed favorably
Park N (2022)^[12]^	46	Diffuse abdominal pain with intermittent obstructive symptoms	Colonoscopy detected a huge mass in the mid-ascending colon	Laparoscopic right hemicolectomy with lymph node dissection was performed	The patient recovered well
Abdulla HA (2020)^[13]^	51	Generalized colicky abdominal pain, vomiting, constipation and abdominal distension	Laboratory investigations showed leucocytosis, CT scan of the abdomen showed small bowel obstruction by a well-circumscribed, intraluminal hypodense mass with fat attenuation in the ileocaecal junction measuring 2.5 × 1.8 cm	Emergency laparotomy	No significant complications postoperatively
Bokhari SFH (2022)^[14]^	68	Severe abdominal pain, nausea, vomiting, and constipation	Computed tomography (CT) scan of the abdomen and pelvis was done, which confirmed small bowel obstruction. A well-circumscribed, intraluminal mass with radiolucent center was observed at the ileocecal junction measuring 2.5 × 1.5 cm	Emergency laparotomy	Patient was hemodynamically stable and afebrile.
Atri S (2024)^[15]^	44	Right lower quadrant pain persisting for 12 h, without passage of stool, gas, or vomiting	Laboratory tests showed leucocytosis, CT scan, which revealed distension of the ileal loops with hydro-aerial content, with a maximum measured diameter of 32 mm with Caecal lipoma measuring 25 × 27 × 40 mm	Right hemicolectomy with ileocolostomy at the right lower quadrant was performed	Continuity was restored after 3 months

**Table 1:** Initial laboratory evaluation

Investigation	Result	Reference range
Hemoglobin	12 g/dL	13–17
Total leukocyte count	7.5 × 10^3^ cells/µL	4–10 × 10^3^
Platelets	263 000 cells/µL	150–450
Serum Sodium	136 mEq/L	135–145
Serum potassium	4.1 mEq/L	3.5–5.0
Serum chloride	111 mEq/L	98–108
Serum TSH	1.2 mIU/L	0.5–5
Serum calcium	8.2 mg/dL	8–10
Blood Urea	36 mg/dL	8–24
Serum creatinine	1.1 mg/dL	<1.2
ALT	39 IU/L	10–50
ALP	112 IU/L	<130
Total bilirubin	1.1 mg/dL	<1.2
HBsAg	Negative	
Anti HCV (IgG)	Negative	
Stool for occult blood	Positive	

ALT, alanine transaminase; ALP, alkaline phosphatase; HBsAg, hepatitis surface antigen; g/dL, gram per deciliter; mEq/L, milliequivalent per liter; U/L, unit per liter; mg/dL, milligram per deciliter.

Imaging methods play a crucial role in diagnosing intussusception. Abdominal ultrasonography is cost-effective, widely accessible, noninvasive, and effective in diagnosing intussusception, particularly when a palpable mass is present. On ultrasound, a lipoma appears as a round, echogenic mass, with the “target or doughnut sign” visible in the transverse view and the “pseudo kidney sign” in the longitudinal view indicating intussusception. However, ultrasound has limitations, such as being operator-dependent and challenging to interpret in the presence of air, which is common in cases of intestinal obstruction^[3,14,15]^. A CT scan is the most reliable method for diagnosing intussusception, offering up to 100% sensitivity and specificity. The classic CT findings include the “target” sign or “sausage-shaped” soft tissue mass, along with mesenteric vessels around the lumen of the intestinal loop. Colonoscopy can confirm the presence of intussusception, identify the lead point, and also serve as a therapeutic option. However, it is important to note that preoperative diagnosis can be challenging in some cases^[16]^.

The treatment for intussusception in adults is almost always surgical, determined by factors such as the size and location of the lipoma, preoperative diagnostic confirmation, and the presence of complications^[17,18]^. Most experts recommend surgically resecting lipomas larger than 2 cm, particularly in older patients, as intussusception in this group is more often associated with malignancy. Unlike in children, nonoperative reduction with barium or air is not typically recommended in adults due to the likelihood of an underlying pathological lead point or predisposing disease^[14,18]^.

While there is no universal approach, many experts advocate for laparotomy due to the high likelihood of a pathological lesion^[17]^. Opinions differ on whether to perform primary resection or attempt reduction before resection. Small bowel lesions are often considered less likely to be malignant; thus, if a preoperative diagnosis indicates a benign lesion and en bloc resection poses a risk of short bowel syndrome, reduction may be a viable option, provided the bowel is not ischemic. However, this must be balanced against potential complications, such as bowel rupture, anastomotic leaks, or tumor seeding^[17]^.

For older patients with colonic involvement, formal oncologic resection followed by anastomosis between healthy, viable bowel is generally recommended. Given the increased risk of malignancy, adhering to oncological principles during resection is crucial. Surgical options typically include segmental resection or, when malignancy is suspected, more extensive bowel resection. If bowel viability is uncertain or if gangrenous bowel is present, immediate resection without reduction is advised to prevent contamination^[17-19]^.

Consequently, surgical resection is considered the optimal treatment for intussusception in adults. Additionally, endoscopic removal of lipomatous polyps and laparoscopic removal of benign small intestinal tumors causing intussusception are other potential treatment methods^[14,18]^.

## Conclusion

A giant lipoma in the terminal ileum can serve as a significant leading point for ileocecal intussusception. This condition’s clinical presentation can be variable, often manifesting as chronic constipation or can be incidentally found during a routine colonoscopy. The pivotal role of imaging in the accurate diagnosis of this condition cannot be overstated, as it helps in identifying the intussusception and its underlying cause. Surgical intervention remains the definitive treatment, aimed at excising the leading point and resolving the intussusception. Early recognition and appropriate surgical management are crucial to prevent potential complications and ensure favorable patient outcomes.

## Data Availability

The datasets supporting the conclusions of this article are included within the article.

## Research registration unique identifying number (UIN)

Researchregistry (researchregistry10571).
